# Long‐Term Outcome of Electrochemotherapy in the Treatment of Advanced Stages (T_3_ –T_4_ ) of Feline Nasal Planum Squamous Cell Carcinoma

**DOI:** 10.1111/vco.70087

**Published:** 2026-07-03

**Authors:** Delia Franchini, Ilaria Laino, Chiara Perrone, Antonio Di Bello, Stefano Ciccarelli

**Affiliations:** ^1^ Department of Veterinary Medicine University of Bari Aldo Moro Valenzano Bari Italy

**Keywords:** advantage stages, electrochemotherapy, palliative therapy, squamous cell carcinoma

## Abstract

Electrochemotherapy is increasingly used as a locoregional treatment for feline squamous cell carcinoma, but its role in advanced nasal planum disease remains poorly defined. This retrospective study evaluated the long‐term outcome of ECT as a sole treatment modality in 18 cats with T_3_–T_4_N_0_M_0_ nasal planum squamous cell carcinoma. Adverse events were assessed according to VCOG‐CTCAE v2. Response was assessed using RECIST v1.1 criteria, and progression‐free survival (PFS), overall survival (OS) and time to progression (TTP) were analysed using Kaplan–Meier methods. At the end of treatment, 72.2% of cats achieved complete response, 16.7% partial response and 11.1% stable disease, resulting in an overall response rate of 88.8%. The 1‐year complete response rate was 61.1%. Treatment response was significantly associated with PFS (*p* = 0.006), OS (*p* < 0.001) and TTP (*p* = 0.006), while recurrence was associated with shorter PFS (*p* = 0.003) and OS (*p* = 0.002). The 1‐year risk of recurrence was 50%. Adverse events were mostly low‐grade (VCOG Grade I–II) and self‐limiting, the most common being transient local pain, erythema/crusting and pruritus; no grade ≥ 3 complications such as tissue necrosis or fistula formation were observed. These results suggest that ECT is an effective and well‐tolerated treatment also in advanced stages nasal planum squamous cell carcinoma, with high response rates and long‐term control disease. The risk of recurrence underscores the need for prolonged follow‐up, as additional treatment sessions may be performed upon relapse, while ECT can also be used with palliative intent to prolong survival while preserving quality of life.

## 1 Introduction

Feline squamous cell carcinoma (fSCC) is a malignant tumour that accounts for approximately 15% of all cutaneous neoplasms in cats and typically affects older cats and primarily arises in unpigmented or lightly pigmented skin [1]. Lesions most commonly involve the nasal planum, eyelids and pinnae [1, 2, 3]. The tumour is characteristically locally aggressive, whereas metastases are uncommon and usually limited to regional lymph nodes and lungs [2, 4]. Feline cutaneous SCC in staged according to the World Health Organization (WHO) TNM classification system (Table 1) [4].

**TABLE 1:** TNM classification system for feline epidermal tumours according to WHO [4].

Stage	Feature
T	
T_0_	No evidence of tumour
T_is_	Tumour in situ
T_1_	Tumour < 2 cm in diameter
T_2_	Tumour 2–5 cm in diameter or minimally invasive
T_3_	Tumour > 5 cm in diameter or with invasion of subcutis
T_4_	Tumour invading other structures such as fascia, muscle or bone
N	
N_0_	Absence of lymph node metastasis
N_1_	Presence of lymph node metastasis
M	
M_0_	Absence of distant metastasis
M_1_	Presence of distant metastasis

Several treatment approaches have been described for fSCC, depending on tumour location and extent [1, 5], including surgery [1, 6, 7], cryotherapy [8], radiotherapy [1, 9, 10], brachytherapy [11], electrochemotherapy (ECT) [12], photodynamic therapy [13] and chemotherapy [14]. To date, surgical excision represents the standard approach, offering the longest disease‐free intervals [6]. However, in advanced‐stage tumours (T_3_–T_4_), achieving tumour‐free margins often requires highly aggressive and potentially disfiguring procedures, which may result in significant functional impairment and may not be acceptable to owners [1, 2]. In this context, increasing attention has been directed toward ECT as a potential alternative therapeutic option, owing to its relative accessibility, repeatability and favourable balance between efficacy and treatment‐related morbidity [3]. Overall, treatment is generally well tolerated, with most reported adverse effects being mild and self‐limiting, including local inflammation, oedema and alopecia [2, 15, 16]. However, in more advanced tumours, more severe complications, including tissue necrosis, fistula formation, pain and functional impairment secondary to scarring, have been reported [12, 15, 17, 18].

Most available studies on ECT have included heterogeneous populations, with variability in tumour location, stage and treatment protocols and in many cases ECT has been combined with other therapeutic modalities [3, 5, 7, 14, 18]. Even when ECT has been specifically evaluated in locally advanced nasal planum SCC, as in the recent series of 21 cats reported by Ferrer‐Jordà and Rodríguez‐Pizà [2], the study population included both treatment‐naïve and previously treated cats, with a follow‐up of approximately 6 months. Consequently, the role of ECT as a single treatment modality, particularly in advanced‐stage disease, remains ill‐defined and its long‐term outcomes, durability of tumour control and safety profile in this setting have not been adequately characterised.

The aim of the present study was to evaluate ECT as a single treatment modality in cats with advanced‐stage (T_3_–T_4_) nasal planum fSCC. By focusing on a homogeneous population and incorporating long‐term follow‐up, we aimed to assess its effectiveness in terms of disease control, as well as its potential role as a palliative option.

## 2 Materials and Methods

### 2.1 Patients and Staging

This retrospective study included owned cats diagnosed with T_3_–T_4_N_0_M_0_ nasal planum SCC treated with ECT at the Department of Veterinary Medicine‐University of Bari between January 2021 and June 2025. The study was approved by the ‘Ethics Committee for Animal Experimentation’ (CESA—‘Animal Welfare Body’ number protocol 31/21).

Inclusion criteria required a cytological or histopathological diagnosis of nasal planum SCC, absence of regional lymph node and distant metastases, use of ECT as the sole treatment modality, with no additional oncologic therapies administered and a minimum follow‐up period of 1 year. Cats with T_1_–T_2_ SCC, other SCC locations, incomplete treatment or follow‐up data and concurrent systemic diseases (including other neoplastic conditions) were excluded.

Data retrieved from medical records included breed, sex, age, tumour location and presenting clinical signs. Diagnostic work‐up comprised complete blood count, serum biochemistry profile, cytological (FNA) or histological evaluation (2‐mm punch biopsy), FNA of regional lymph nodes and thoracic radiographs (three projections) for staging according to the WHO classification (Table 1) [4]. Additional information included feline immunodeficiency virus (FIV) and feline leukaemia virus (FeLV) status and pre‐ and post‐ECT treatment photographs.

### 2.2 Treatment and Management

The ECT treatment was performed with Onkodisruptor (Biopulse, Rome, Italy), an electric pulse generator, under general anaesthesia. The anaesthetic protocol included premedication with dexmedetomidine 10 μg/kg (Cepedex; P.H. Farmaceutici) and ketamine 2 mg/kg (Ketabel; P.H. Farmaceutici) administered intramuscularly; induction with fentanyl 2 μg/kg (Fentadon; Dechra) and propofol 2–4 mg/kg (PROPOMITOR; Ecuphar) administered intravenously, followed by patient intubation. Anaesthesia maintenance was achieved through the administration of isoflurane (IsoFlo, Zoetis, Italy).

Bleomycin (Bleoprim 15 000 UI; Sanofi, Italy) was administered at a dose of 15 mg/m^2^ iv in bolus. Eight minutes after bleomycin administration, trains of 8 biphasic pulses (50 + 50 μs, 10 μs interphase delay) were delivered via a Stick Probe handpiece with interchangeable two‐needle (45 mm) and two‐plate (1 cm [2], with conductive gel) electrodes, at 1 Hz and 1200 V/cm.

The number of ECT sessions was determined according to clinical evaluation. In cats with persistent macroscopic disease and an unsatisfactory clinical response at re‐evaluation, an additional ECT session was proposed once adequate local healing had occurred. The interval between ECT sessions was based on clinical response, local tissue recovery and clinician assessment.

Post‐treatment care included the use of an Elizabethan collar for at least 5 days to prevent self‐trauma at the ECT site; meloxicam (Meloxoral; Dechra Veterinary Products S.r.l.) at 0.1 mg/kg on Day 1, followed by 0.05 mg/kg orally for the subsequent 3 days; and/or gabapentin (Gabapentin; DOC Generici S.r.l., Italy) at 10 mg/kg twice daily (BID), according to pain assessment based on VCOG criteria [19]. Specific antibiotic therapy was administered only in cases of secondary infection or lesions resulting from self‐trauma. In cats with reduced appetite, transdermal mirtazapine ointment was administered to minimise the risk of anorexia [20].

### 2.3 Outcome

Treatment response was assessed according to RECIST v1.1 criteria [21], and categorised as complete response (CR), partial response (PR), stable disease (SD) or progressive disease (PD). Evaluations were performed weekly during the first month after ECT, monthly during the first 6 months, and every 3 months thereafter until the end of the observation period (June 2025) through clinical examination and photographic documentation. Final treatment response was defined as the response recorded 4 weeks after the last ECT session.

Overall response rate (ORR) was defined by the percentage of cats achieving CR or PR. Overall survival (OS) was defined as the time from first ECT to death [22]. Progression‐free survival (PFS) was defined as the time from first ECT until first evidence of disease progression or death [22]. Time to progression (TTP) was defined as the time from first ECT until first evidence of disease progression [23].

### 2.4 Toxicity

Adverse events of ECT were assessed according to VCOG‐CTCAE, v.2 [19]. Pruritus, alopecia, erythema, skin ulcerations, tissue necrosis, pain and systemic symptoms (such as anorexia and lethargy) were assessed.

### 2.5 Statistics

Data were analysed using inferential statistical methods. Survival analysis was performed using the Kaplan–Meier method, and a log‐rank test was performed to assess differences in OS, PFS, and TTP according to treatment response (CR, PR, SD and PD), stage (T_3_ vs. T_4_), and retroviral status. Recurrence was evaluated in relation to OS and PFS. Patients lost to follow up or alive at the time of analysis were censored. The cumulative risk of recurrence within 1 year after treatment was estimated as 1 − *S*(*t*), where *S*(*t*) was derived using the Kaplan–Meier method. A Fisher's exact test was used to assess the association between the type of ECT (reversible vs. irreversible) and the occurrence of recurrence.

Statistical significance was set at *p* < 0.05. Statistical analyses were performed using SPSS software (version 27.0.1.0; IBM Corp., Armonk, NY, USA).

## 3 Results

Eighty‐three cats with fSCC were treated with ECT during the study period. Thirty‐eight cats were excluded due to lesions located at other sites (pinnae, eyelids, or lips). Of the 45 cats diagnosed with nasal planum SCC, 11 were classified as T_1_N_0_M_0_ and 10 as T_2_N_0_M_0_. Two cats classified as T_3_N_1_M_0_ and 4 as T_4_N_1_M_0_ were excluded due to lymph node involvement. A total of 18 domestic short‐haired cats met the inclusion criteria and were enrolled. The median age was 10.9 years (range: 10–14 years). All cats were neutered, including 55.6% males (*n* = 10) and 44.4% females (*n* = 8). Baseline clinical signs included abnormal upper airway noises, such as stridor, in 83.3% of cats (*n* = 15), sneezing in 77.8% (*n* = 14), pain in 67% (*n* = 12) and anorexia or reduced appetite in 44.4% (*n* = 8). At initial clinical evaluation, 38.9% (*n* = 7) of cats exhibited marked nasal obstruction and respiratory wheezing. In 88.9% (*n* = 16), the neoplastic process involved both nostrils, resulting in facial deformity and alteration of the nasal planum profile (Figures 1 and 2).

**FIGURE 1:**
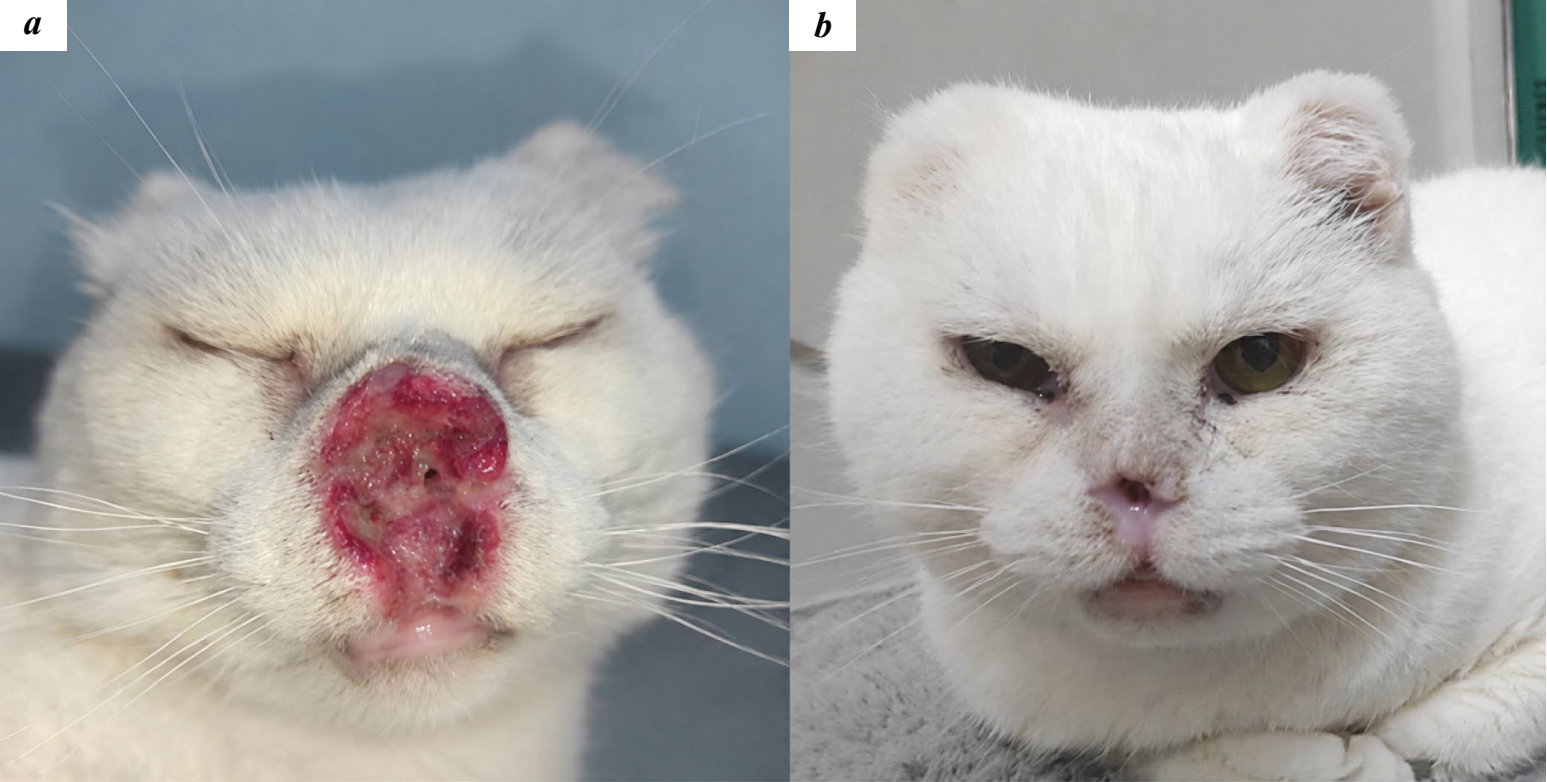
(a) Cat 6 (T_4_N_0_M_0_) at presentation and (b) the patient in CR 600 days after treatment.

**FIGURE 2:**
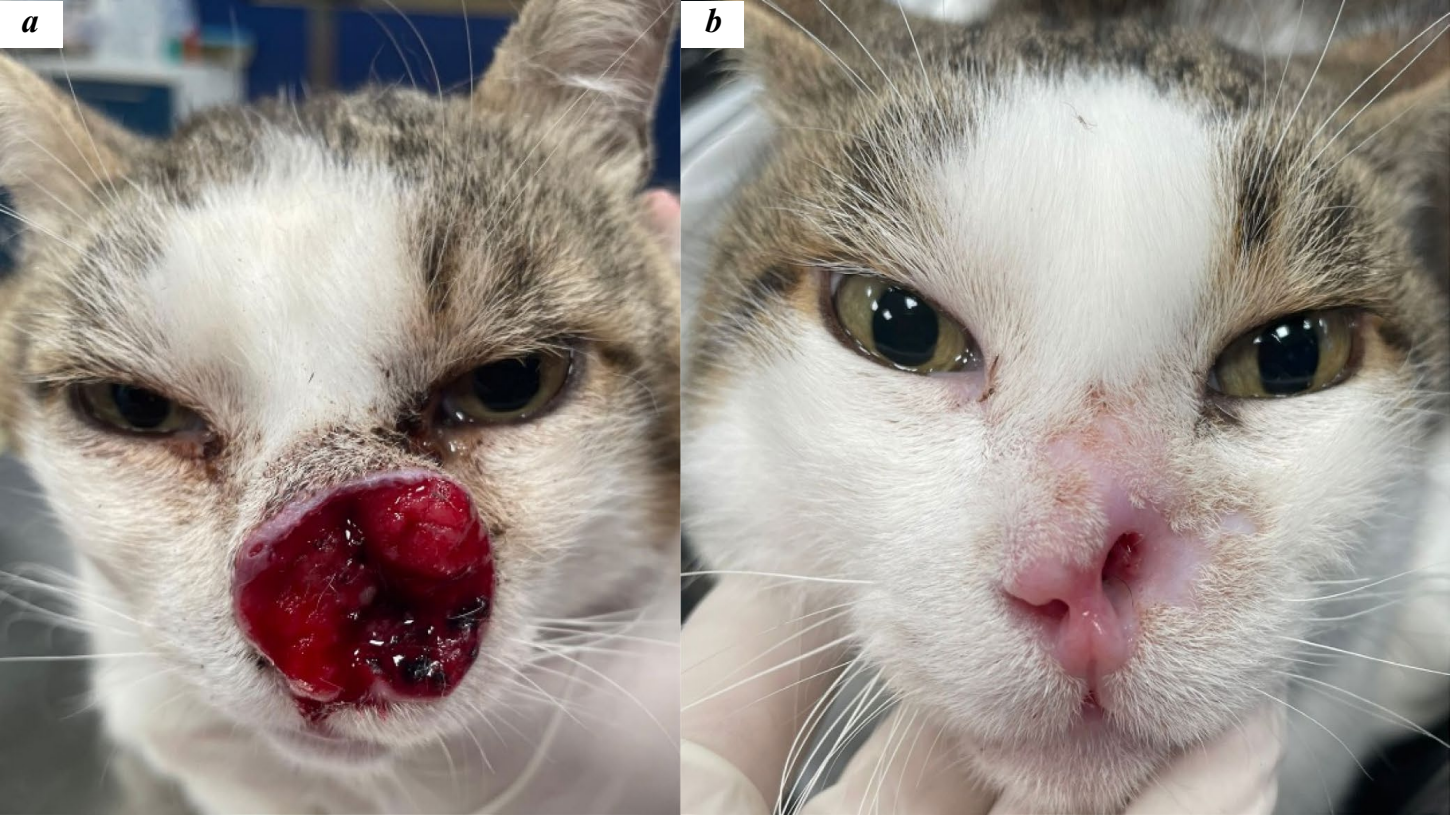
(a) Cat no. 14 (T_4_N_0_M_0_) at presentation and (b) the patient in CR 380 days after treatment.

In remaining 11.1% (*n* = 2) of cats, the disease was confined to either the right or left nostril.

Diagnosis was obtained by cytology in 83.3% (*n* = 15) of cats and biopsy in 16.7% (*n* = 3). Ten cats (55.6%) were staged as T_3_N_0_M_0_ and 8 (44.4%) as T_4_N_0_M_0_; two cats (11.1%) were FIV‐positive (Table 2).

**TABLE 2:** Summary of signalment, retroviral status, staging, number of ECT, response at the end of treatment, therapy and survival for all cases included in the study.

Cat	Signalament	FIV/FeLV	Who stage	Number of treatments	Response to therapy	TTP	PFS	OS	Subsequent therapies	Survival
1	MN, 11Y	FIV+	T_4_N_0_M_0_	3	SD	151	151	368	NSAIDs, gabapentin, antibiotic, mitarzapine	Dead, progressive disease
2	FN, 10Y	FIV/FeLV−	T_4_N_0_M_0_	2	CR	/	/	720	NSAIDs, gabapentin	Alive, still CR
3	MN, 14Y	FIV/FeLV−	T_4_N_0_M_0_	1	SD	65	65	370	NSAIDs, gabapentin, antibiotic, mitarzapine	Dead, progressive disease
4	MN, 10Y	FIV/FeLV−	T_4_N_0_M_0_	2	CR	365	365	608	NSAIDs, gabapentin	Alive, progressive disease
5	FN, 11Y	FIV/FeLV−	T_4_N_0_M_0_	3	CR	/	390	390	NSAIDs, gabapentin	Dead, unreleated cause
6	FN, 13Y	FIV/FeLV−	T_4_N_0_M_0_	1	CR	/	690	690	NSAIDs, gabapentin	Dead, unreleated cause
7	FN, 13Y	FIV/FeLV−	T_3_N_0_M_0_	2	CR	/	820	820	NSAIDs	Dead, unreleated cause
8	FN, 13Y	FIV/FeLV−	T_3_N_0_M_0_	2	PR	35	35	366	NSAIDs, gabapentin	Dead, progressive disease
9	FN, 10Y	FIV/FeLV−	T_3_N_0_M_0_	1	CR	/	/	1390	NSAIDs	Alive, still CR
10	MN, 11Y	FIV/FeLV−	T_3_N_0_M_0_	3	CR	/	/	1559	NSAIDs, gabapentin	Alive, still CR
11	MN, 13Y	FIV+	T_4_N_0_M_0_	2	PR	56	56	374	NSAIDs, gabapentin	Dead, progressive disease
12	FN, 12Y	FIV/FeLV−	T_3_N_0_M_0_	2	CR	/	/	481	NSAIDs, gabapentin	Alive, still CR
13	FN, 12Y	FIV/FeLV−	T_3_N_0_M_0_	1	CR	120	120	373	NSAIDs	Dead, progressive disease
14	FN, 12Y	FIV/FeLV−	T_4_N_0_M_0_	2	CR	/	/	365	NSAIDs, gabapentin	Alive, still CR
15	MN, 10Y	FIV/FeLV−	T_3_N_0_M_0_	1	CR	260	260	640	NSAIDs, gabapentin	Dead, progressive disease
16	MN, 13Y	FIV/FeLV−	T_3_N_0_M_0_	1	CR	/	/	365	NSAIDs	Alive, still CR
17	FN, 12Y	FIV/FeLV−	T_3_N_0_M_0_	1	CR	/	/	365	NSAIDs, gabapentin	Alive, still CR
18	FN, 10Y	FIV/FeLV−	T_3_N_0_M_0_	2	CR	/	/	473	NSAIDs, gabapentin	Alive, still CR

Abbreviations: FeLV = feline leukaemia virus, FIV = feline immunodeficiency virus, FN = female neutered, MN = male neutered, OS = overall survival, PFS = progression‐free survival, TTP = time to progression, Y = years.

Overall, 38.9% of cats (*n* = 7) received one ECT session, 44.4% (*n* = 8) received two sessions and 16.7% (*n* = 3) received three sessions. Second and third ECT sessions were proposed in cats with persistent macroscopic disease, as assessed at re‐evaluation approximately 4 weeks after the previous ECT session. The mean interval between the first and second sessions was 30.8 days (range, 21–64 days), and the mean interval between the second and third sessions was 26.7 days (range, 18–40 days).

After the first ECT session, PR was observed in 55.6% of cats (*n* = 10) and CR in 33.3% (*n* = 6), while SD and PD were each observed in one cat (5.6% each). Among cats receiving a second session, 54.5% (*n* = 6) achieved CR, 36.4% (*n* = 4) had PR and 9.1% (*n* = 1) had SD. After the third session, 66.7% of cats (*n* = 2) achieved CR and 33.3% (*n* = 1) had SD.

At the end of treatment, 72.2% of cats (*n* = 13) achieved CR, 16.7% (*n* = 3) achieved PR and 11.1% (*n* = 2) had SD, yielding an ORR of 88.8%. After 1 year, 61,1% (*n* = 11) of cats were still in CR.

Three cats were retreated during the observation period due to recurrence. One cat staged T_4_N_0_M_0_ received three ECT sessions. After the first session, the cat showed PD, and after the second and third sessions the disease remained stable. After 151 days disease progression was then observed, and two additional palliative ECT sessions were administered. Due to PD, the cat was euthanised, resulting in an OS of 368 days. One T_3_N_0_M_0_ cat underwent a single ECT session and achieved CR; recurrence occurred at 120 days and retreatment again resulted in CR. A second recurrence after 223 days progressed to PD, and the cat was euthanised 1 month later, resulting in an OS of 373 days. Another T_3_N_0_M_0_ cat received a single ECT session and achieved CR; recurrence occurred at Day 260 and retreatment again resulted in CR. A further recurrence developed 284 days later, progressed to PD, and the cat was euthanised 96 days thereafter, resulting in an OD of 640 days.

At the end of the observation period, 50% of the cats were still alive. Nine cats died during the study period, including six euthanised due to tumour progression, two following the development of renal disease, and one following the development of cardiac disease (Table 3).

**TABLE 3:** Adverse events graded according to VCOG‐CTCAE v2 [21] observed after electrochemotherapy (ECT) sessions, reported as grade, percentage, and number of affected cats.

ECT session	Adverse event	Grade	Percentage (%)	*N*/total
First	Pruritus	1	27.8	5/18
	Erythema and crusting	2	33.3	6/18
	Pain	2	72.2	14/18
	Alopecia	1	5.5	1/18
	Anorexia	2	5.5	1/18
Second	Pruritus	1	37.5	3/8
	Erythema and crusting	1	62.5	5/8
	Pain	1	12.5	1/8
Third	Alopecia	1	33.3	1/3
	Pruritus	1	33.3	1/3
	Anorexia and lethargy	2	33.3	1/3

Adverse events (VCOG‐CTCAE v.2) [19] observed after ECT sessions are summarised in Table 3.

Two cats staged T_4_N_0_M_0_ required antibiotic therapy and transdermal mirtazapine.

Kaplan–Meier survival curves are shown in Figures 3 and 4 and only statistically significant comparisons based on the log‐rank test are presented. The median PFS for the cohort was 690 days, the median OS was 640 days and the median TTP was not reached.

**FIGURE 3:**
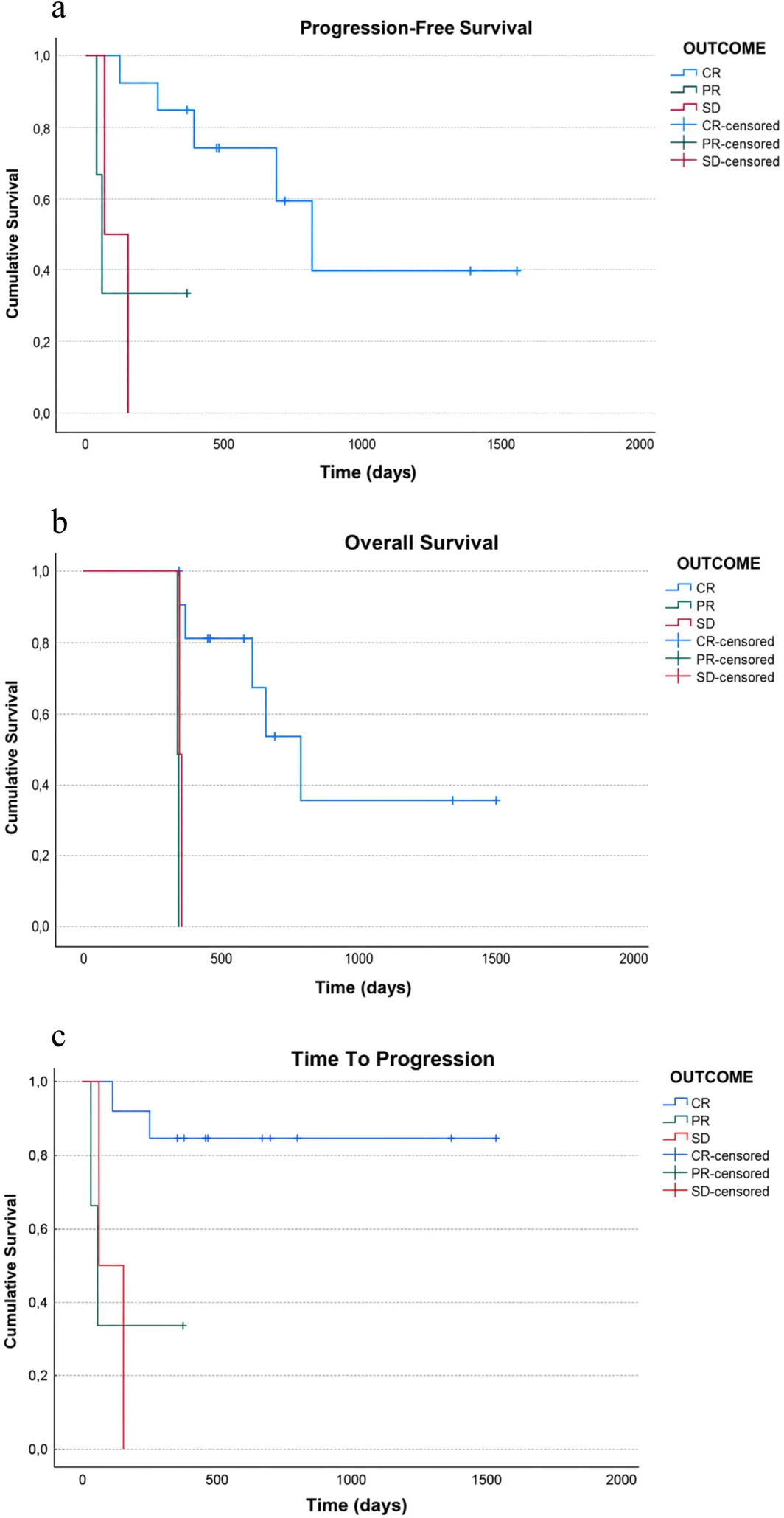
(a) Kaplan–Meier progression‐free survival stratified by response group (CR, PR, SD); tick marks denote censored cases. A significant difference among groups was detected (log‐rank test, *p* = 0.006). (b) Kaplan–Meier overall survival by response group (CR, PR, SD); tick marks denote censored cases (log‐rank test, *p* < 0.001). (c) Kaplan–Meier time‐to‐progression stratified by response group (CR, PR, SD); tick marks denote censored cases (log‐rank test, *p* = 0.006).

**FIGURE 4:**
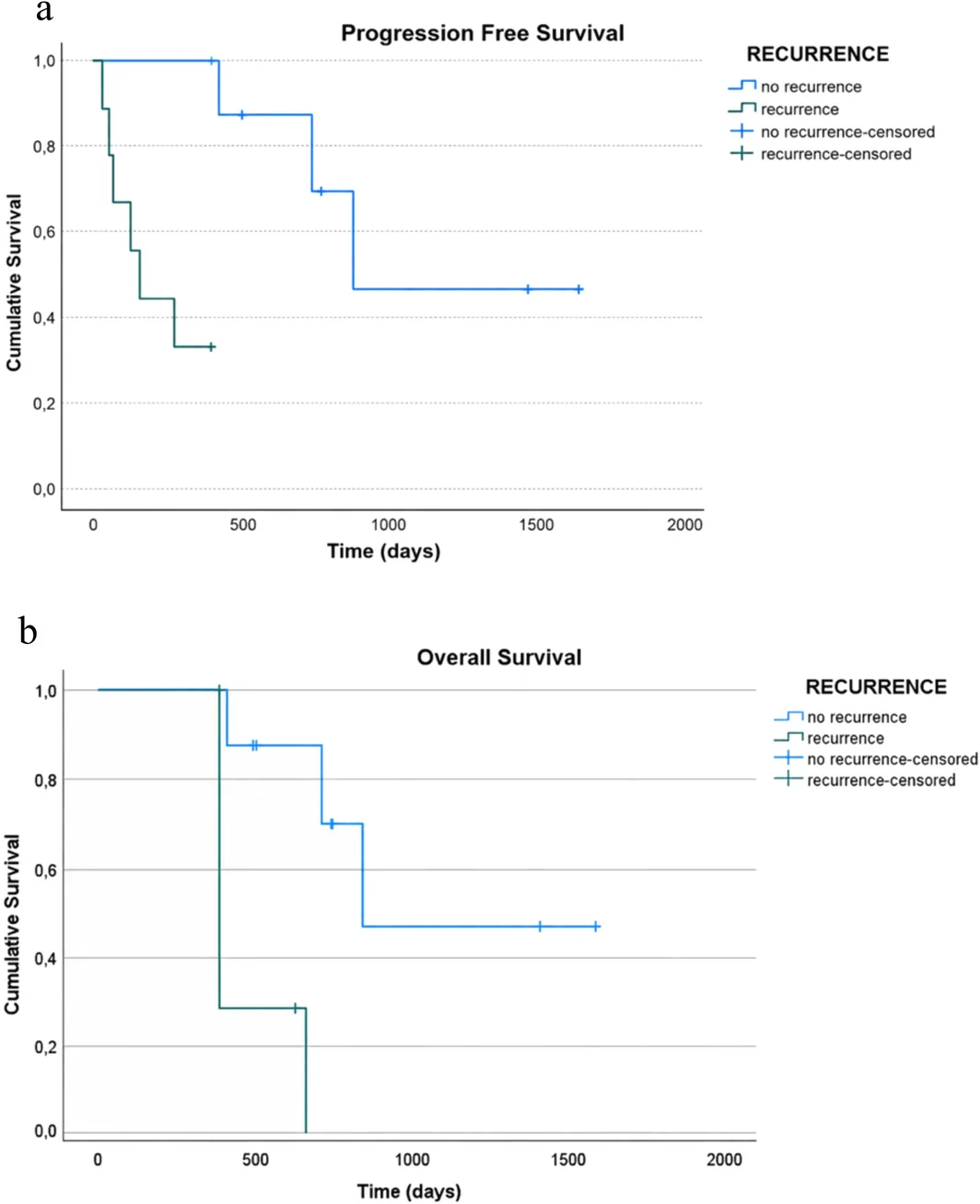
(a) Kaplan–Meier progression‐free survival stratified by recurrence; tick marks denote censored cases. A significant difference between groups was detected (log‐rank test, *p* = 0.003). (b) Kaplan–Meier overall survival stratified by recurrence; tick marks denote censored cases (log‐rank test, *p* = 0.002).

Treatment response appeared to be associated with clinical outcome in this cohort. Cats were stratified according to response as CR (*n* = 13), PR (*n* = 3) and SD (*n* = 2). Significant differences were observed for PFS (*p* = 0.006), OS (*p* < 0.001) and TTP (*p* = 0.006). Median PFS was 820 days in cats achieving CR, 56 days in cats with PR and 65 days in cats with SD. Median OS was 820 days in cats achieving CR, 366 days in cats with PR and 368 days in cats with SD. Recurrence status was also associated with outcome. Cats were stratified as having recurrent disease (*n* = 6) or non‐recurrent disease (*n* = 12). Cats with recurrence had shorter PFS (*p* = 0.003) and OS (*p* = 0.002) than cats without recurrence. Median PFS was 820 days in cats without recurrence, compared with 151 days in cats with recurrence. Median OS was 820 days in non‐recurrent cats and 373 days in cats with recurrence. No statistically significant associations were observed for the other variables analysed. The 1‐year risk of recurrence was 50%. Fisher's exact test revealed no significant association between session type and the probability of recurrence.

## 4 Discussion

The present study evaluated ECT as a single treatment modality in cats with advanced‐stage (T_3_–T_4_) nasal planum SCC, with long‐term follow‐up. Our findings suggest that ECT can achieve high response rates even in advanced‐stage disease, with complete remission in treated patients and durable disease control in a subset of cases, supporting its potential role in the management of locally advanced fSCC.

Although ECT has been widely investigated in fSCC, interpretation of published outcomes is often limited by substantial heterogeneity in tumour stage, anatomical location, metastatic status, and treatment protocols [2, 3, 5, 7, 18, 24]. In contrast, the present study focused on a homogeneous population of cats with advanced‐stage nasal planum SCC without metastasis, all treated using a consistent ECT protocol. Within this more strictly defined clinical setting, the achievement of a CR rate of 72.2% and an ORR of 88.8% suggests that ECT may remain effective even in the presence of advanced local disease. Comparable CR rates have been reported in feline ECT series that included earlier‐stage tumours, namely 77.7% by Spugnini et al. in T_2_–T_4_ disease and 65.3% (ORR 78.3%) by Foo et al. [18] across T_1_–T_4_ stages; the rate observed in our exclusively advanced‐stage cohort is therefore particularly noteworthy.

These rates are all the more relevant because they were obtained in tumours that, by definition, exceed the size threshold associated with reduced ECT efficacy. In a meta‐analysis of single‐session ECT, the CR rate fell to approximately one‐third for tumours ≥ 3 cm, compared with around 60% for smaller lesions [25], and tumour size has likewise been identified as a determinant of local response in feline nasal planum SCC [5]. The 72.2% CR rate achieved in our T_3_–T_4_ cohort therefore exceeds what size‐based models would predict. A plausible explanation lies in the treatment schedule: further CRs were obtained after the second (*n* = 6) and third (*n* = 2) sessions, in keeping with the proposal that large tumours may require repeated ECT cycles to achieve adequate control [25]. These observations reinforce the view that tumour burden or clinical stage alone do not fully determine the response to ECT [5, 18, 25]. While direct comparisons across studies should be interpreted with caution, our results further support the role of ECT as a valuable locoregional treatment option in this context, particularly when radiation therapy, although considered a standard approach [10], is limited by cost, the need for repeated general anaesthesia, and restricted availability in many clinical settings, including Italy, where radiation therapy is currently available at a single referral centre.

Among the available studies, the report by Ferrer‐Jordà and Rodríguez‐Pizà is particularly relevant, as it specifically evaluated cats with T_3_–T4 nasal planum SCC treated with ECT [2]. In that cohort, 90.5% of cats achieved CR, and the ORR was 100%, compared with 72.2% and 88.8%, respectively, in the present study. Median OS was 453 days, whereas median OS was 640 days in our population. While these numerical differences should be interpreted cautiously, both studies support the efficacy of ECT in locally advanced disease and demonstrate its ability to achieve meaningful tumour control in a substantial proportion of patients. Important methodological differences should also be considered when comparing the two studies. Ferrer‐Jordà and Rodríguez‐Pizà included both treatment‐naïve and previously treated cats, and additional rescue therapies such as surgery and toceranib were administered after recurrence in some cases. In contrast, cats included in the present study received ECT as the sole oncologic treatment modality throughout the study period. Furthermore, the minimum follow‐up required for inclusion was longer in the present study, allowing a more comprehensive assessment of recurrence patterns and long‐term outcome. Consequently, the two studies should be viewed as complementary rather than directly comparable, collectively strengthening the evidence supporting ECT for the management of advanced feline nasal planum SCC.

In the present cohort, the number of ECT sessions and the interval between treatments were not uniform, but were adapted to the clinical evolution of each case. Although 33.3% of cats (6/18) achieved CR after the first session, additional treatments were required in the remaining cases to obtain tumour control, with further CR observed after the second (*n* = 6) and third session (*n* = 2). The interval between sessions was broadly in line with previously reported clinical practice [3, 6], but was not standardised, as retreatment was tailored to individual response and clinical condition. In this setting, retreatment decisions were influenced not only by the persistence of residual disease, but also by the need to allow recovery from local tissue reactions and tumour‐related clinical signs. While it might be expected that the need for multiple sessions simply reflects tumour stage, our data do not support a clear stage‐dependent pattern. Although the limited sample size precluded a statistical comparison between T_3_ and T_4_ groups, early CR was also observed in some T_4_ tumours, whereas not all T_3_ lesions showed a more favourable response pattern. This suggests that tumour stage alone may not fully explain variability in treatment response.

Other factors, including the true local extent and individual clinical presentation, may have contributed to the variability observed. In particular, the lack of advanced imaging (CT or MRI) may have limited precise assessment of tumour extension, potentially influencing both treatment planning and the timing and extent of response. In addition, although no association was identified between retroviral status and clinical outcome, the limited number of FIV‐positive cases precludes any definitive conclusions, and a potential influence on treatment response cannot be entirely excluded. This variability indicates that treatment response cannot be reliably predicted based on initial tumour presentation. Nevertheless, once achieved, response had a strong impact on clinical outcome, with markedly longer PFS and OS in cats achieving CR (median 820 days for both) compared with those with PR (56 and 366 days, respectively) or SD (65 and 368 days, respectively). These findings are consistent with previous reports identifying treatment response as a key prognostic factor [2, 7].

Recurrence emerged as a significant prognostic factor, being associated with markedly shorter survival compared with non‐recurrent cases (median PFS: 151 vs. 820 days; median OS: 373 vs. 820 days). However, beyond its impact on outcome, the temporal pattern of recurrence provides additional insight into the clinical behaviour of the disease.

Notably, although 61.1% (11/18) remained disease‐free at 1 year, the estimated probability of recurrence during the first year remained 50%, highlighting that recurrence may occur even after a prolonged period of initial disease control. From a clinical perspective, these data emphasise that follow‐up should not be limited to the early post‐treatment period. While close monitoring during the first year remains essential, continued surveillance beyond this time point is equally critical to identify late recurrence. Importantly, the occurrence of recurrence does not preclude further intervention, as additional treatments may allow re‐establishment of disease control and contribute to prolonged survival and maintenance of quality of life. In this context, ECT may be considered not only as a definitive approach in selected cases, but also as a repeatable palliative strategy.

ECT was generally well tolerated, with most adverse events being mild and transient. Local pain, erythema, and pruritus were the most frequently observed findings, and only a limited number of cases required additional medical management.

However, many cats already presented with clinically relevant signs at baseline, including pain and respiratory discomfort, which were transiently exacerbated following treatment but subsequently improved or resolved, supporting a positive impact on quality of life. This pattern suggests that, particularly in advanced disease, clinical signs are more likely driven by tumour burden rather than by treatment‐related toxicity. The transient worsening observed after treatment may be attributed to an acute local inflammatory response induced by ECT, temporarily exacerbating pre‐existing tumour‐associated inflammation and pain, and should therefore be interpreted within the context of overall disease control [17].

This study has several limitations, including its retrospective design and relatively small sample size, which limited statistical power and precluded subgroup analyses. Advanced imaging (CT or MRI) was not performed, which may have limited a more accurate assessment of local tumour extension. Finally, the number and timing of ECT sessions were not standardised but adapted to individual clinical evolution, reflecting real‐world practice but introducing heterogeneity. Finally, the number and timing of ECT sessions were not standardised but adapted to individual clinical evolution, reflecting real‐world practice but introducing heterogeneity.

## 5 Conclusions

ECT represents an effective and well‐tolerated treatment option for cats with advanced‐stage nasal planum SCC, achieving high response rates and durable disease control even in locally advanced disease. Outcomes comparable to those reported in less advanced or heterogeneous populations suggest that tumour stage alone may not preclude response. Clinical management was primarily guided by treatment response and disease evolution over time, supporting a dynamic, individualised approach. The occurrence of late recurrences highlights the need for prolonged follow‐up and further supports the role of ECT as a repeatable palliative strategy, capable of restoring disease control while preserving quality of life.

## Funding

The authors have nothing to report.

## Conflicts of Interest

The authors declare no conflicts of interest.

## Data Availability

The data that support the findings of this study are available from the corresponding author upon reasonable request.
